# Recursive Indirect-Paths Modularity (RIP-M) for Detecting Community Structure in RNA-Seq Co-expression Networks

**DOI:** 10.3389/fgene.2016.00080

**Published:** 2016-05-09

**Authors:** Bahareh Rahmani, Michael T. Zimmermann, Diane E. Grill, Richard B. Kennedy, Ann L. Oberg, Bill C. White, Gregory A. Poland, Brett A. McKinney

**Affiliations:** ^1^Tandy School of Computer Science, University of TulsaTulsa, OK, USA; ^2^Division of Biomedical Statistics and Informatics, Department of Health Sciences Research, Mayo ClinicRochester, MN, USA; ^3^Mayo Clinic Vaccine Research Group, Mayo ClinicRochester, MN, USA; ^4^Department of Mathematics, University of TulsaTulsa, OK USA

**Keywords:** sequence analysis, RNA, gene expression profiling, newman modularity, weighted gene correlation network analysis, WGCNA, algorithms

## Abstract

Clusters of genes in co-expression networks are commonly used as functional units for gene set enrichment detection and increasingly as features (attribute construction) for statistical inference and sample classification. One of the practical challenges of clustering for these purposes is to identify an optimal partition of the network where the individual clusters are neither too large, prohibiting interpretation, nor too small, precluding general inference. Newman Modularity is a spectral clustering algorithm that automatically finds the number of clusters, but for many biological networks the cluster sizes are suboptimal. In this work, we generalize Newman Modularity to incorporate information from indirect paths in RNA-Seq co-expression networks. We implement a merge-and-split algorithm that allows the user to constrain the range of cluster sizes: large enough to capture genes in relevant pathways, yet small enough to resolve distinct functions. We investigate the properties of our recursive indirect-pathways modularity (RIP-M) and compare it with other clustering methods using simulated co-expression networks and RNA-seq data from an influenza vaccine response study. RIP-M had higher cluster assignment accuracy than Newman Modularity for finding clusters in simulated co-expression networks for all scenarios, and RIP-M had comparable accuracy to Weighted Gene Correlation Network Analysis (WGCNA). RIP-M was more accurate than WGCNA for modest hard thresholds and comparable for high, while WGCNA was slightly more accurate for soft thresholds. In the vaccine study data, RIP-M and WGCNA enriched for a comparable number of immunologically relevant pathways.

## Introduction

Modularity is a property of many complex systems where the components of the system are organized into functional subunits or modules. Modular organization can be observed in engineered systems like hardware components of a computer, packages in software, or parts of a vehicle. But modularity is also observed in evolved systems like DNA into chromosomes, spatial regions of the brain into specialized functions, or genes into transcriptional regulation networks (Shen-Orr et al., 2002). It is commonly held that cellular organization and biochemical function is modular in nature (Hartwell et al., 1999; Mitra et al., 2013). There are likely multiple selective pressures that lead to modularity in evolved systems, one of which may be the frequency of a changing environment (Parter et al., 2007). Intuitively, if an environment is relatively static, an evolving system has the luxury to build large modules, whereas in a rapidly changing environment, there is a greater advantage to building and reusing smaller, robust functional subunits (modules).

RNA-Seq is a next-generation sequencing technology for the genome-wide quantification of gene expression that is rapidly growing in prevalence and has some advantages over microarrays in characterizing transcriptomes (Ozsolak and Milos, 2011; Hitzemann et al., 2013). There are also many challenges to analyzing RNA-Seq data and few studies have investigated the network properties of RNA-seq based co-expression networks (Ballouz et al., 2015). One of the perennial statistical challenges of clustering data-driven networks (e.g., gene co-expression) is how optimally to determine the appropriate number and size of clusters. Hierarchical clustering relies on the relative distance between genes or samples, represented as trees. Clustering can be described as “cuts” of these trees, which can result in single clusters that encompass the majority of genes, or conversely, many small (potentially singleton) clusters. Accordingly, one of the motivations of the Weighted Gene Correlation Network Analysis (WGCNA) strategy is to avoid small clusters, enabled by parameters to specify a minimum module size and merge cut height (Langfelder et al., 2013). Model-based clustering uses likelihood and model-complexity to guide the user to an appropriate number of clusters and may be put into a hierarchical framework (Guo et al., 2010), but the size of the clusters may still have extremes.

Newman Modularity is a spectral clustering algorithm that performs multiple binary splits of the network and determines cluster memberships by optimizing a quadratic-form of the difference between observed and expected connections (under null model assumptions). Submodules are found automatically through recursive binary splits (Newman, 2006). Automated cluster number estimation is an attractive feature of modularity (data-driven); however, when applied to the RNA-Seq co-expression networks in the current study, modularity identifies a small number of large components that encompass the bulk of genes (e.g., **Figure 8**). To uncover the biologically relevant substructures, larger modules must be split and smaller modules merged in an optimal way.

The implicit goal of spectral clustering is to find a balance between groups in order to find more realistic communities and avoid trivial solutions (Bolla, 2011). For example, the potential for trivial solutions led to the proposal of normalized cut penalties to avoid minimal cut clusters that contain a single node separated from the rest of the nodes (Shi and Malik, 2000). In the case of gene co-expression networks, large modules may lead to the enrichment of functional categories that are too broad to be biologically informative for the given study. For example, when we use various settings of a hard threshold, Newman Modularity results in modules with over 1000 genes. Such large clusters should be further resolved to map onto more specific functional categories. At the same time, very small clusters may need to be broadened to identify statistically significant overlap with functional biological categories.

In the current study, we develop a generalized indirect-paths form of modularity and incorporate it into a merge-and-split algorithm we call “recursive indirect-paths modularity” (RIP-M). The RIP-M algorithm varies the order of finite power series of the adjacency matrix, which adaptively merges small clusters by incorporating indirect path evidence for module membership. Recent studies using powers of the adjacency matrix include subgraph centrality (Estrada and Rodriguez-Velazquez, 2005) and network deconvolution (Feizi et al., 2013). The subgraph centrality method used a sum of local moments of the adjacency matrix powers to derive a new centrality algorithm. Local moments use the diagonal of each matrix power. In our approach, we compute a modified modularity, as opposed to centrality, and we use the diagonal and off-diagonal of the power series up to a finite order. Also, our algorithm adapts the power series order to change the size and number of clusters. Network deconvolution reduces the noise in a network by assuming an infinite power series of the direct network, which allows one to invert the observed network to reverse the effect of transitive information flow from the theoretical indirect paths. Our approach adapts the order of the power series in subnetworks (modules) to reduce and magnify transitive effects within and between subnetworks in the observed network and thereby modify the size and number of clusters.

Including higher powers of the adjacency matrix increases the flow of information between a given pair of genes based on shared indirect connections, which, along with recursive splitting, allows RIP-M to develop modules with sizes near a user defined range. The goal of the RIP-M algorithm is to provide a user with the flexibility to specify a “Goldilocks” range that penalizes module sizes from being too large or too small for the biological questions being asked. We compare the accuracy of RIP-M with Newman modularity and WGCNA on simulated co-expression networks with homogeneous and heterogeneous cluster sizes. We compare the accuracy of methods for hard and soft thresholds, and we compare the methods based on functional enrichment of relevant pathways on real RNA-Seq data.

## Methods

In this section, we describe our new community detection algorithm, RIP-M, and our evaluation strategy based on simulated co-expression data and real data from an RNA-Seq experiment of an influenza vaccine study. Our aim is to detect communities or clusters of genes that are related by correlation across subjects in a gene expression study. To construct the network, we begin with a matrix of Pearson correlation coefficients (−1 ≤ ρ_*ij*_ ≤ 1) between the expression levels of genes i and j. We then define a binary adjacency matrix *A*_*ij*_ ∈ {0, 1} by the condition |*A*_*ij*_| ≥ τ, where τ is a threshold. The value of τ is one factor that influences the size distribution of modules. We discuss the selection of τ and soft thresholds below.

### Recursive indirect-paths modularity (RIP-M)

Our RIP-M algorithm is a modification of Newman Modularity. RIP-M iteratively merges and splits modules to obtain a desired range of module sizes. If a module is larger than a user-defined threshold, the module is split based on iteration of modularity to this submodule. Merging is based on the application of Modularity to a power series of the adjacency matrix, which encodes indirect paths between nodes and has the effect of reassorting nodes into larger modules. We first review the relevant properties of Modularity.

#### Newman Modularity

Modules of an adjacency matrix, *A*_*ij*_, are determined by recursive binary partitions of the nodes in the network. The base of the recursive algorithm is the binary partition of the network. The partition is encoded in a vector *s*_*i*_ ∈ {−1, 1}, where *s*_*i*_ = 1 if node *i* is in the first community and *s*_*i*_ = −1 if node *i* is in the second community. The vector s is obtained by maximizing the modularity Q:

(1) $$\begin{array}{l} Q=\frac{1}{4m}\underset{ij}{\sum }\left(A_{ij}-\frac{k_{i}k_{j}}{2m}\right){\;s}_{i}s_{j}, \end{array}$$

where *k*_*i*_, *k*_*j*_ are the degree of nodes *i* and *j*, and $m=\frac{1}{2}\underset{i}{\;\sum }k_{i}$. The quantity *k*_*i*_*k*_*j*_/2*m* is the expected number of edges between the nodes *i, j* for an Erdos-Renyi random network. We may rewrite *Q* in a quadratic matrix form as:

(2) $$\begin{array}{l} Q=\frac{1}{4m}s^{T}B\;s, \end{array}$$

where the matrix $B_{ij}=\left(A_{ij}-\frac{k_{i}k_{j}}{2m}\right)$. In order to maximize the modularity efficiently, one relaxes the requirement that the elements of *s* be dichotomous and one computes *s* as the dominant eigenvector of B. The values *s* are then dichotomized into 1 and −1 by taking the sign of the elements of the dominant eigenvector. This algorithm determines the initial binary partition of the network and is used recursively to form additional partitions. Within a subgroup of nodes *g* in a partition, the change in Q, Δ*Q*, is computed by maximizing *B*^(*g*)^:

(3) $$\begin{array}{l} \Delta Q=\frac{1}{4m}s^{T}B^{\left(g\right)}s,\;B^{\left(g\right)}=\underset{i,j\in g}{\sum }\left[B_{ij}-\delta_{ij}\underset{k\in g}{\sum }B_{ik}\right] \end{array}$$

where *B*^(*g*)^ is the subgroup modularity matrix properly accounting for the degree of nodes prior to binary splitting. Recursive partitioning stops when Δ*Q* is sufficiently small.

#### Generalized (indirect-paths) modularity (IP-M)

We can generalize Equations (1–3) by writing Q as a function of a path matrix or power series of the adjacency matrix to order n:

(4) $$\begin{array}{l} P^{\left(n\right)}=A+A^{2}+\cdots +A^{n}. \end{array}$$

For a binary (non-weighted) adjacency matrix, an element of *P*^(*n*)^ represents the number of paths with length smaller or equal to n between nodes i and j. This transforms the input network for modularity to a network weighted by indirect connections. For example, two nodes may not be directly connected, but multiple shared nodes may suggest that the two nodes should be in the same community. We find that this has a merging effect on communities. Then we can write the generalized-paths modularity as:

(5) $$\begin{array}{l} Q\left(P^{\left(n\right)}\right)=\frac{1}{4m^{\left(n\right)}}\sum_{ij}\left({\left(P^{\left(n\right)}\right)}_{ij}-\frac{k_{i}^{\left(n\right)}k_{j}^{\left(n\right)}}{2m^{\left(n\right)}}\right){\;s}_{i}s_{j}. \end{array}$$

We include the “*n*” superscripts to remind the reader that the degrees are computed for the path matrix P (Equation 4), not the original adjacency matrix A. For a given path order n, the algorithm for computing modularity is the same as Equations (1–3), but using P (Equation 4) instead of A. Next, we describe the RIP-M algorithm, which recursively splits large communities and varies the order n in *Q*(*P*^(*n*)^) (Equation 5) to automatically reassort clusters that are too small. The number of indirect paths (n) adapts within the recursion to attempt to achieve modules in the specified range.

#### RIP-M algorithm

**Figure 1:**
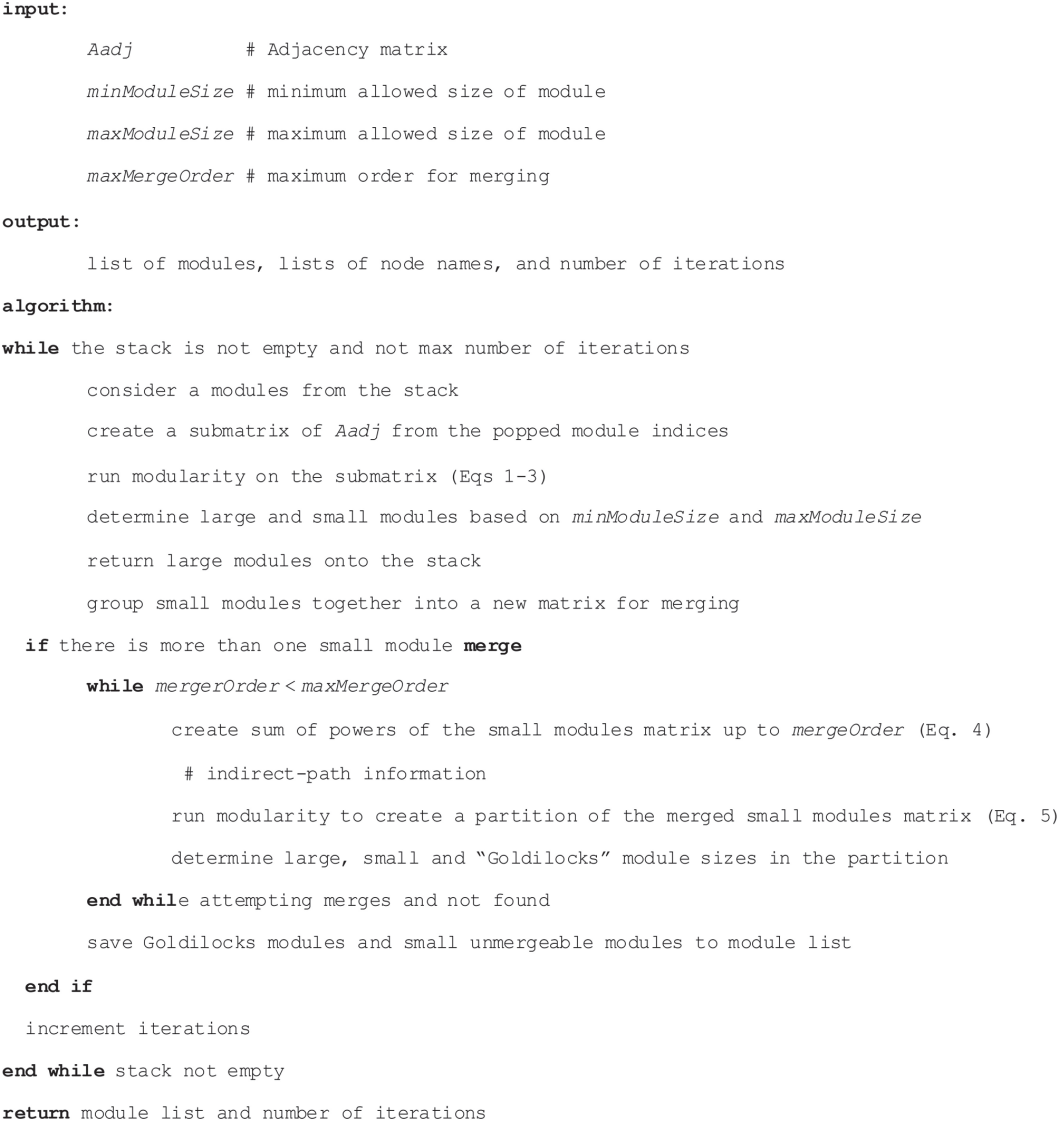
**RIP-M algorithm pseudocode**.

The recursion in RIP-M is implemented as a stack in the R language, described in pseudocode (Figure 1). The first input is the (weighted or un-weighted) adjacency matrix. In our simulation studies, we test hard and soft thresholding of the absolute value of the correlation matrix. A soft threshold is a transformation of the correlation matrix that raises values to a given power, element-wise. In applications, we show that the hard threshold, τ, and the soft-threshold power play important roles in determining the size of clusters. The target range of the module sizes is specified by the parameters minModuleSize and maxModuleSize. Modules below minModuleSize are placed together and fed into generalized modularity for merging with increasing orders of the indirect-paths power series *P*^(*n*)^. The order increases and the merging iteration continues until maxMergeOrder is reached or the module-size target is reached. Modules above maxModuleSize are put in the stack to be split in subsequent iterations. Modules in the target range are added to the return module list.

### Hierarchical WGCNA

Here, we review aspects of the WGCNA method that are relevant for comparison with modularity and the RIP-M method introduced in the current study. WGCNA is a hierarchical clustering method frequently used for clustering gene co-expression networks. It uses agglomerative hierarchical clustering with average linkage, but it uses a dynamic tree cutting to try to balance the size of clusters (Langfelder et al., 2013). This sometimes results in genes that are unassigned to any cluster. WGCNA also includes optional transformations of the correlation matrix, such as hard and soft thresholds and signed and unsigned networks. Parameters for these transformations may be guided by fitting a scale-free degree distribution. An important feature of WGCNA is the Topological Overlap Matrix (TOM) for assigning similarities between genes:

$$t_{ij}=\;\{\begin{array}{cc} \frac{|N_{1}(i)\;\cap \;N_{1}(j)|\;+\;a_{ij}}{min\left\{\left|N_{1}\left(i\right)|,|N_{1}\left(j\right)\right|\right\}\;+\;1-a_{ij}} & if\;i\neq j\\ 1 & if\;i=j \end{array}\;,$$

where |*N*_1_(*i*)| is the number of neighbors of node *i* and *a*_*ij*_ is the corresponding element in the adjacency matrix. This first term in the numerator is the number of genes that genes i and j share as common connections. This term takes advantage of indirect evidence that two genes are similar. The denominator is a normalization factor. WGCNA also includes generalized TOM (GTOM) to perform hierarchical clustering with higher order indirect connections. The TOM matrix is transformed into a distance matrix for hierarchical clustering. We vary GTOM and thresholding parameters to identify the most uniform cluster sizes.

### Methods for cluster comparison

#### Simulated data

We simulate co-expression networks with known partitions or communities, and we compare clustering algorithms based on their accuracy in assigning nodes to their correct partitions. We create 100 replicate simulations for a given set of simulation parameters. We compare the accuracy of each method based on the Rand index (Rand, 1971). For all pairs of nodes, the Rand index determines whether the pair has the same cluster label predicted by the clustering algorithm and compares this concordance with the concordance in the true simulated clusters. We use two simulation strategies, both based on noisy block diagonal structures but with different noise distributions: random uniform and the beta distribution.

In the uniform random strategy, the background of the correlation matrix is composed of random uniform numbers between 0 and r_noise, max_. This matrix is an example of a Wigner matrix (its entries are independent, uniformly bounded random variables, and we force the matrix to be symmetric; Bolla, 2011). On top of the background noise, we superimpose the clusters as block diagonal matrices composed of random uniform numbers between r_signal, min_ and 1. Clusters become more difficult to resolve as the noise and signal limits overlap (i.e., as r_block, min_ becomes larger and/or r_block, max_ becomes smaller). In our simulations, we use highly overlapping parameters (r_noise, max_ = 0.8 and r_signal, min_ = 0.2) to make the community detection more challenging. We force the matrix to be symmetric, but it may not be positive definite.

**Figure 2:**
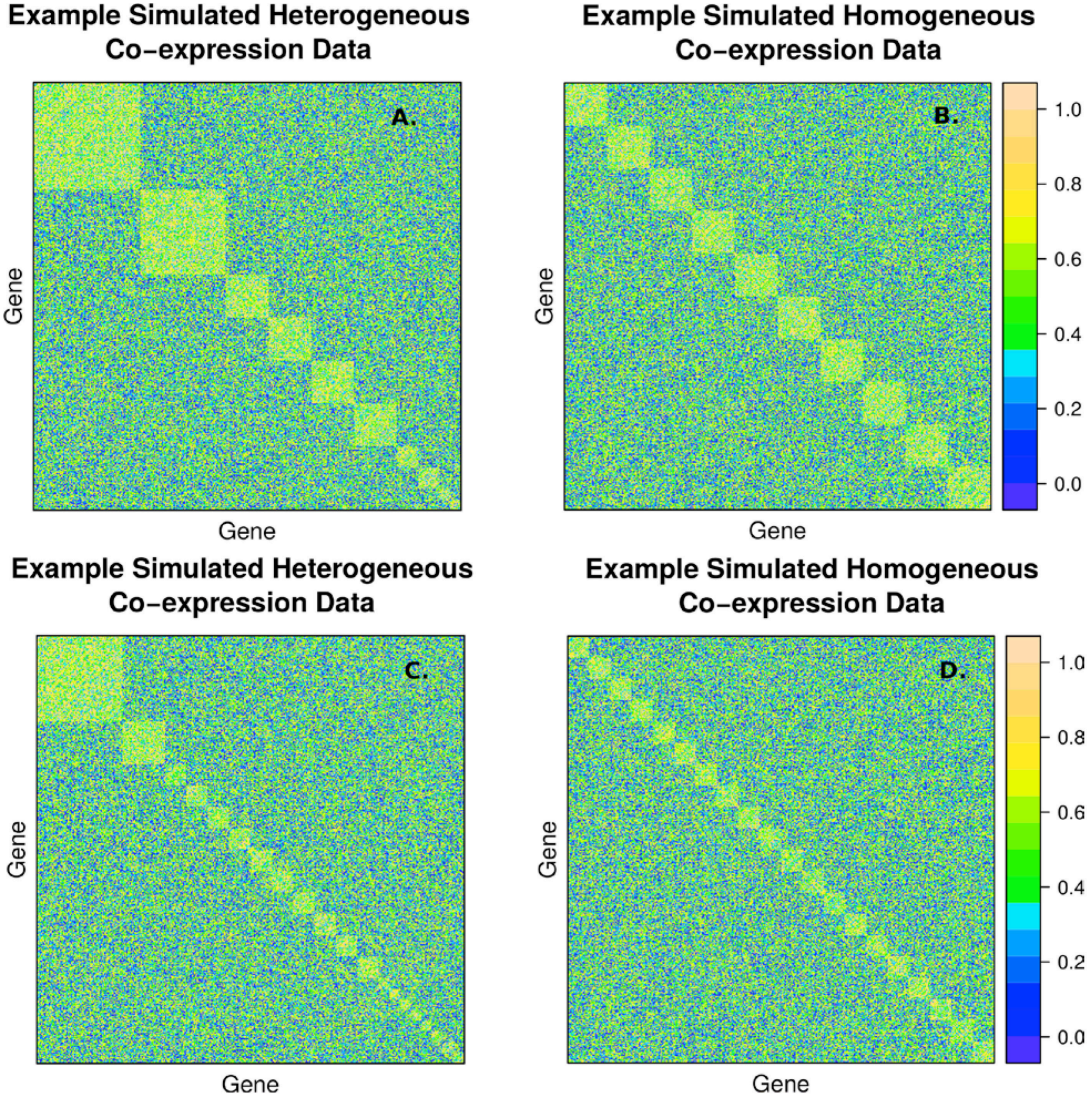
**Example of simulated correlation matrices with 10 clusters (A,B) and 20 clusters (C,D) of 400 nodes with heterogeneous (A,C) and homogeneous (B,D) cluster sizes**. The block diagonal clusters are random uniform numbers between r_signal, min_ and 1. Outside the blocks, the correlations are random uniform numbers between 0 and r_noise, max_. The chosen signal and noise parameters are r_signal, min_ = 0.2 and r_noise, max_ = 0.8, respectively, to simulate a large amount of overlap between signal and background.

**Figure 3:**
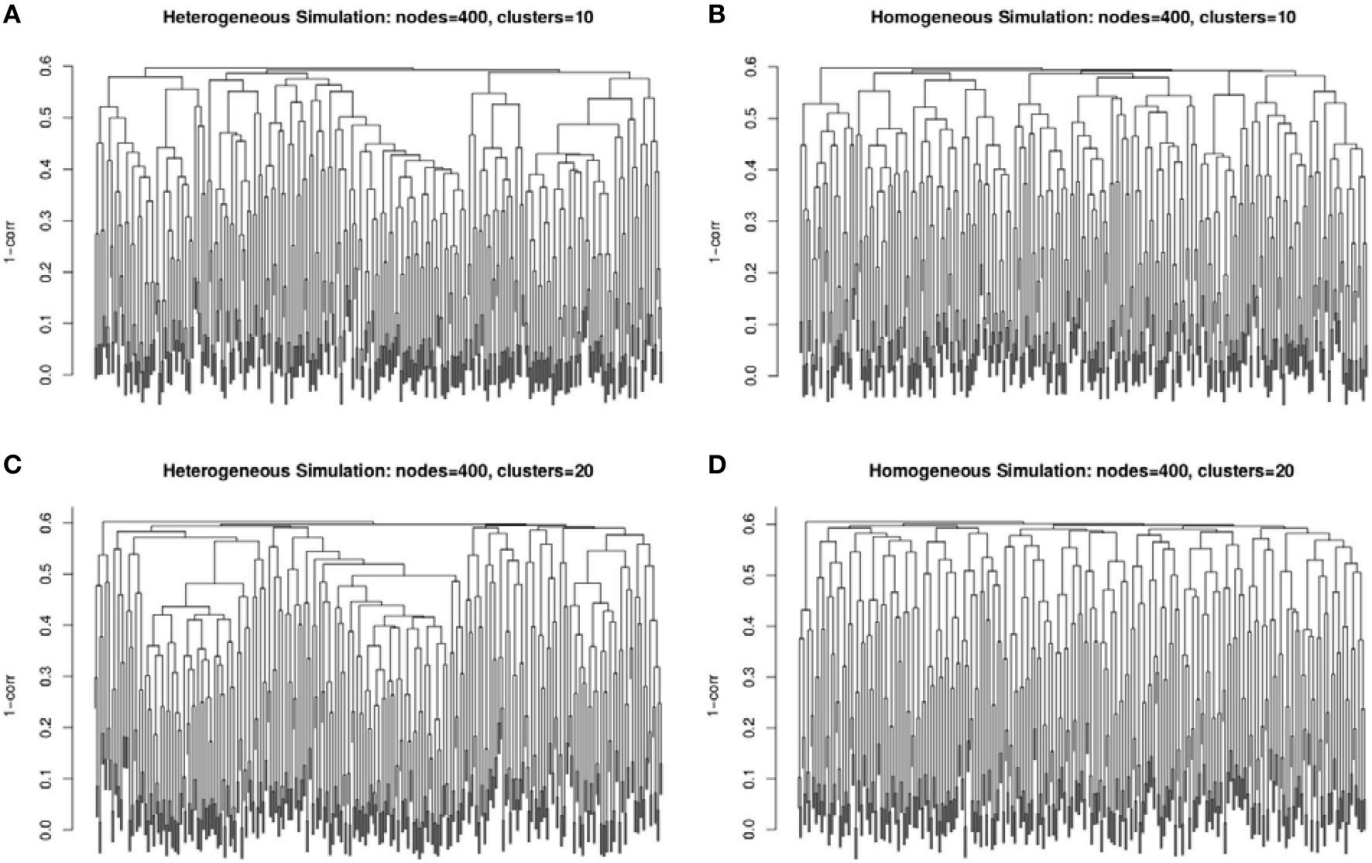
**Example dendrograms from the simulated correlation matrices with 10 clusters (A,B) and 20 clusters (C,D) of 400 nodes with heterogeneous (A,C) and homogeneous (B,D) cluster sizes**. The simulated cluster blocks can be seen in Figure 2.

The second simulation strategy uses a beta distribution with parameters (3, 2) for the signal and (2, 3) for the background noise. These parameters result in similarly challenging signal to noise as in the random uniform strategy, but with a less abrupt transition between clustered genes and non-clustered genes. In addition, we map the final correlation matrix to nearest positive definite matrix (Cheng and Higham, 1998). Since, we expect real co-expression data to display variation in the number and size of clusters, we simulate co-expression networks with heterogeneous (example in Figures 2A,C) and homogeneous (example in Figures 2B,D) cluster sizes. The challenge to cluster detection of the noise parameters can be seen in the flat hierarchy (small distances separating the simulated clusters) in Figure 3.

#### RNA-seq data

RNA-seq measures gene expression by sequencing, yielding the abundance of each transcript present in a sample. We applied each clustering method to RNA-Seq data from an influenza vaccine study of 105 individuals performed at the Mayo Clinic (Rochester, MN). All study participants provided written informed consent, and all study procedures were approved by Mayo Clinic's Institutional Review Board. Gene abundances were computed from transcriptomic sequencing using the MAP-RSeq bioinformatics pipeline tool (Kalari et al., 2014). The Illumin HiSeq 2000 (Illumina, San Diego, CA) sequencing reads were aligned to the human genome build 37.1 using TopHat (1.3.3) and Bowtie (0.12.7). Counting genes and mapping the reads to individual exons was carried by HTSeq (0.5.3p3) and BEDTools (2.7.1), respectively. Total number of counts per gene was obtained from the mRNA expression. Quality control was assessed pre- and post-normalization graphically with minus- vs.-average and box-and-whisker plots. The GC content and gene length adjustments were also evaluated graphically. Normalization of the gene counts was done with Conditional Quantile Normalization (CQN), which accounts for differences in library size and also adjusts for GC content and gene length (Hansen et al., 2012). These normalized values were used for subsequent analyses.

Gene expression levels were measured at Day 0 (before vaccination) and 3 days after vaccination. Clustering was performed on the difference of the log2 CQN normalized data between Day 3 and Day 0. Before clustering, we filtered to the top 4956 transcripts showing the largest variation across timepoints and largest read counts. Due to the perturbation of the gene network by a vaccine, we expect increased activity among immune system pathways. Thus, we used immunological pathways from the Reactome FI database to compare the enrichment from clustering methods of relevant biological pathways for the vaccine immune response experiment (Vastrik et al., 2007). We calculated the fraction of overlap of each module with immunological gene sets and used the hypergeometric *p*-value to quantify significance of enrichment.

## Results

### Simulation results

We compared the average accuracy of Newman Modularity, RIP-M and WGCNA to find the correct number of clusters and the correct cluster identities of all 400 simulated genes based on the Rand index. We simulated 100 replicate datasets of co-expression data simulated with 10 and 20 heterogeneous-sized clusters (e.g., Figures 2A,D, respectively) and 10 and 20 homogeneous-sized clusters (e.g., Figures 2B,C, respectively). We specified a minimum cluster size of 10 and maximum of 50 for the RIP-M and WGCNA algorithm parameters. This range is more of a guideline than a strict rule because cluster sizes routinely go outside the range for both algorithms, and we find that the simulation results are not sensitive to this range. In addition to comparing homogeneous and heterogeneous cluster sizes, we compared methods based on hard and soft thresholding, which are common noise-reduction techniques for co-expression network clustering.

#### Hard threshold results

**Figure 4:**
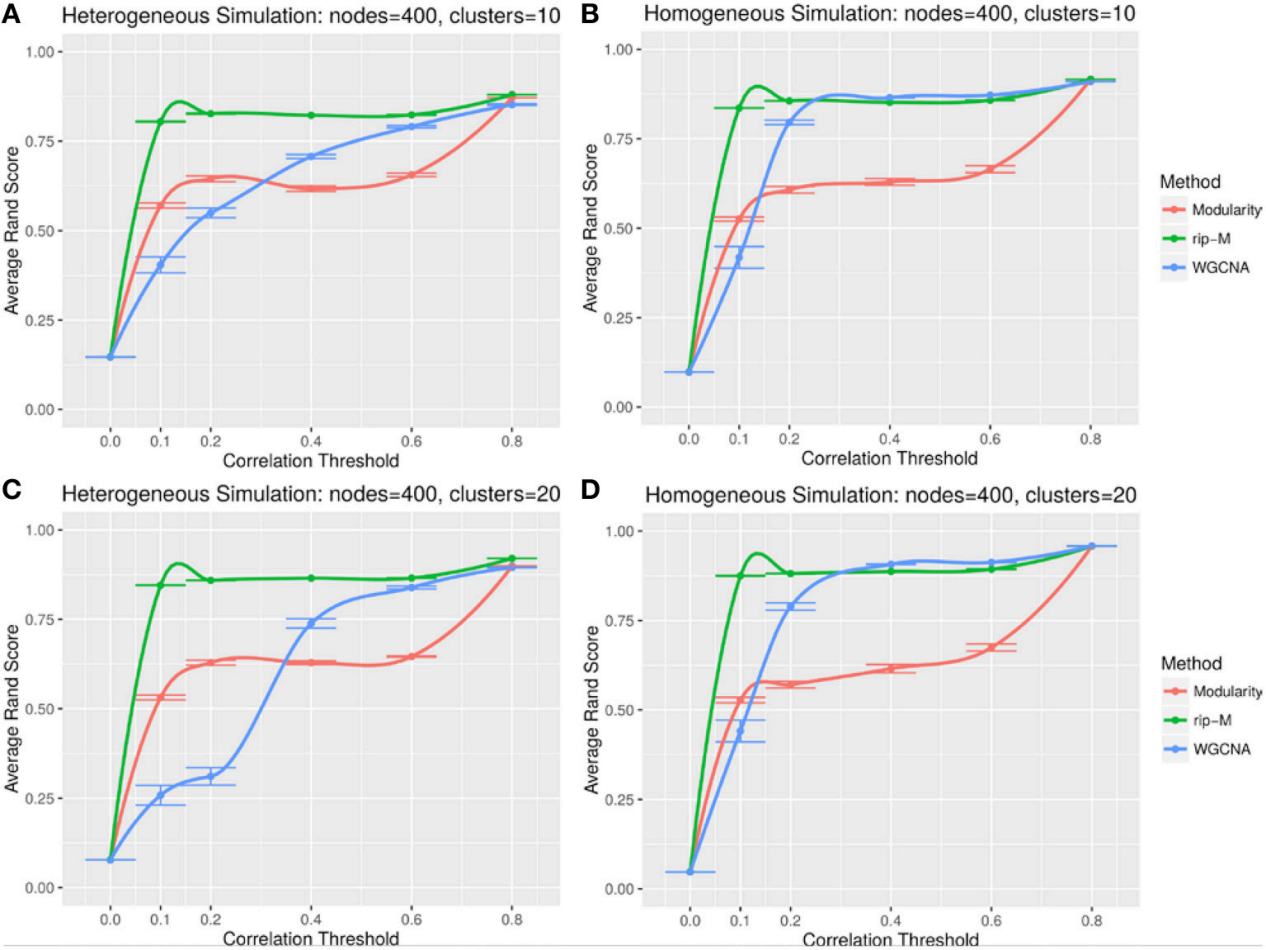
**Comparison of clustering accuracy using hard correlation threshold for random uniform simulated heterogeneous (A,C) and homogeneous (B,D) cluster sizes with 10 clusters (A,B) and 20 clusters (C,D)**. The signal and noise parameters are r_signal, min_ = 0.2 and r_noise, max_ = 0.8, respectively. 100 replicate simulations are created and clustering is carried out for each of the hard threshold cutoffs of the correlation matrix (horizontal axis). The accuracy of the clustering methods (average on the vertical axis) is based on the Rand index of the cluster identities compared with the true simulated cluster identities.

**Figure 5:**
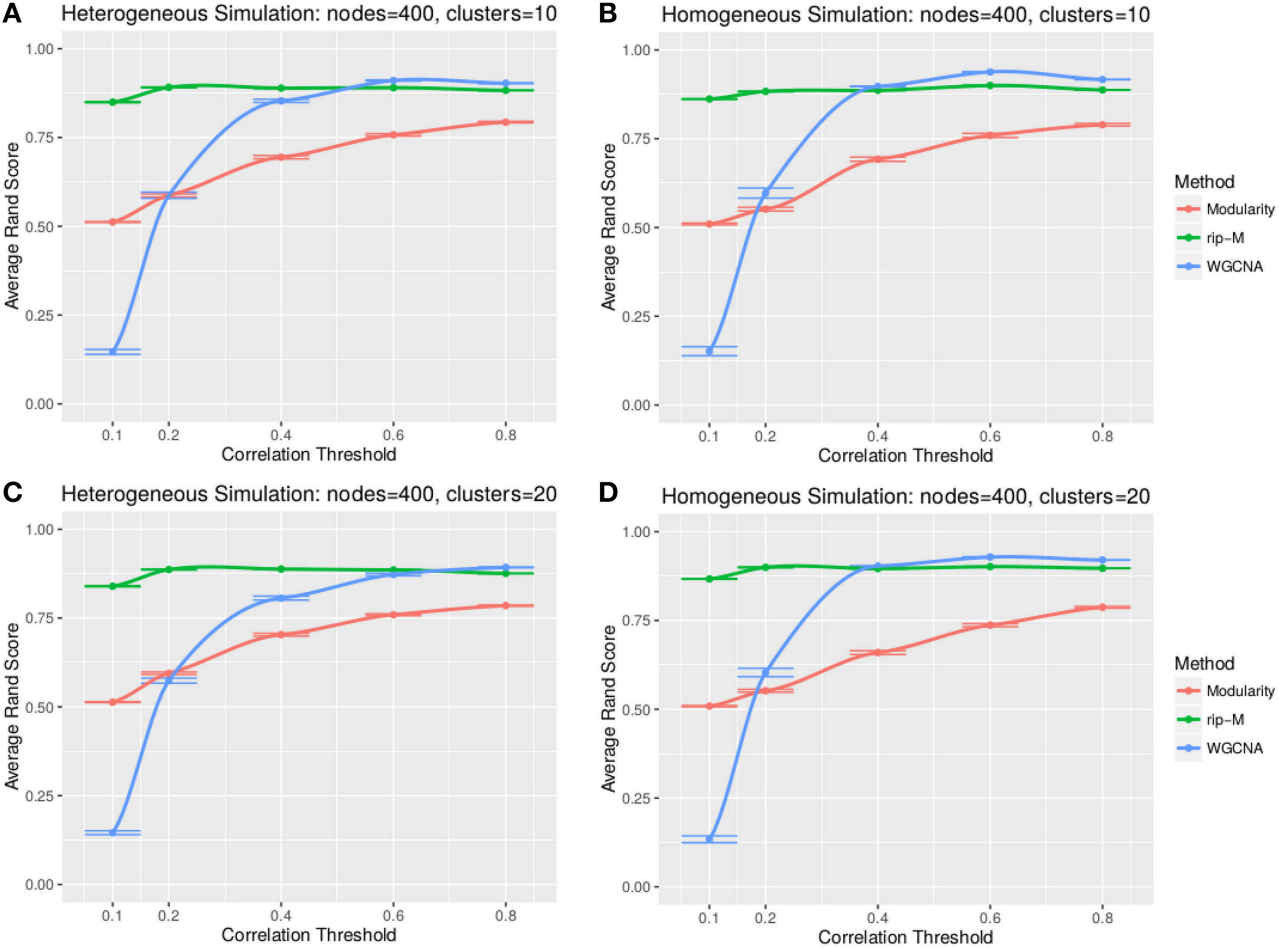
**Comparison of clustering accuracy using hard correlation threshold for beta-distributed noise simulated for heterogeneous (A,C) and homogeneous (B,D) cluster sizes with 10 clusters (A,B) and 20 clusters (C,D)**. The beta noise parameters are (3, 2) for the signal and (2, 3) for the background. One hundred replicate simulations are created and clustering is carried out for each of the hard threshold cutoffs of the correlation matrix (horizontal axis). The accuracy of the clustering methods (average on the vertical axis) is based on the Rand index of the cluster identities compared with the true simulated cluster identities.

A hard threshold yields a binary adjacency matrix with elements equal to 1 when the correlation is above the threshold, and 0 otherwise. The threshold eliminates lower correlations that are more likely to be noise (due to chance). For hard thresholding, RIP-M has a consistently higher accuracy than WGCNA and Newman Modularity across all thresholds and simulation parameters (Figures 4, 5). In the uniform random noise simulation (Figure 4), as the threshold increases, RIP-M reaches its maximum accuracy more quickly than the other methods. When the threshold is 0.8 in the uniform random uniform simulations (Figure 4), all of the methods are able to find the correct clusters with high accuracy because all noise connections are pruned at this threshold. This occurs at this particular threshold because we use r_signal, min_ = 0.2 and r_noise, max_ = 0.8 for the simulated signal and noise parameters, respectively. Many true within-cluster connections are also pruned at the 0.8 threshold, but this does not hinder the algorithms from detecting the community structure. In the simulations with beta-distributed noise (Figure 5), the hard threshold has a smoother effect on the cluster identification accuracy, but the accuracy trends are similar to the uniform random simulation strategies.

#### Soft threshold results

**Figure 6:**
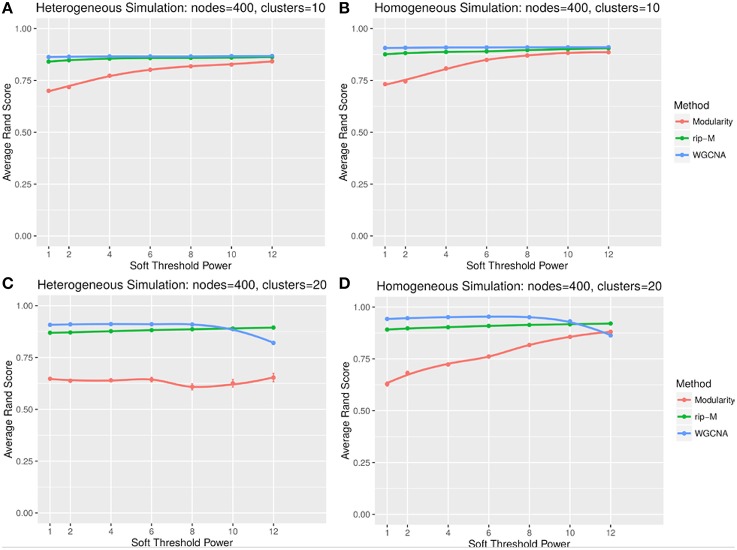
**Comparison of clustering accuracy using soft thresholds for random uniform simulated heterogeneous (A,C) and homogeneous (B,D) cluster sizes with 10 clusters (A,B) and 20 clusters (C,D)**. The signal and noise parameters are r_signal, min_ = 0.2 and r_noise, max_ = 0.8, respectively. One hundred replicate simulations are created and clustering is carried for varying soft threshold powers, which involves taking the element-wise powers of the correlation matrix. The accuracy of the clustering methods (on the vertical axis) is based on the Rand index of the cluster identities compared with the true simulated cluster identities.

**Figure 7:**
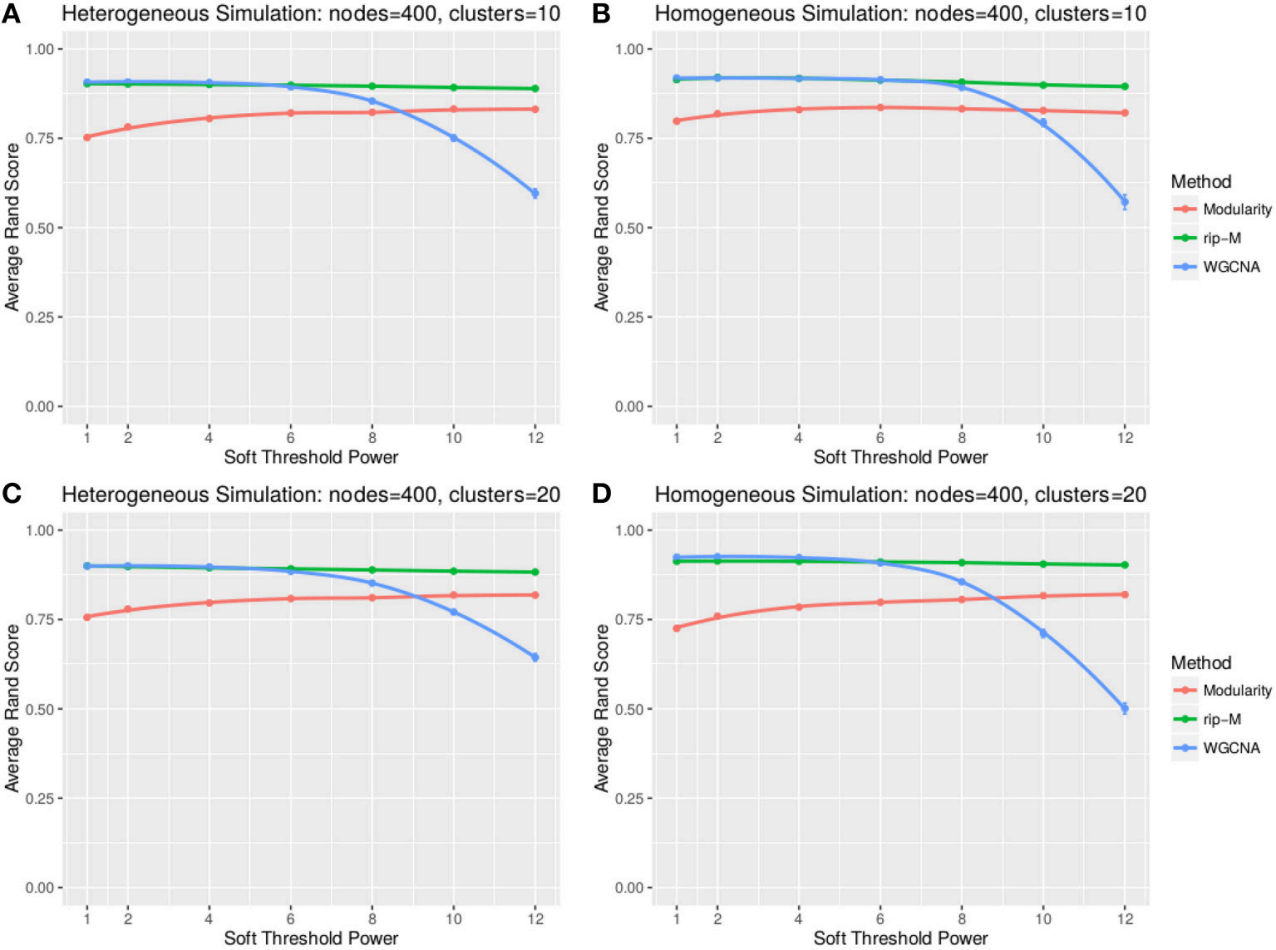
**Comparison of clustering accuracy using soft thresholds for beta-distributed noise simulated for heterogeneous (A,C) and homogeneous (B,D) cluster sizes with 10 clusters (A,B) and 20 clusters (C,D)**. The beta noise parameters are (3, 2) for the signal and (2, 3) for the background. One hundred replicate simulations are created and clustering is carried for varying soft threshold powers, which involves taking the element-wise powers of the correlation matrix. The accuracy of the clustering methods (on the vertical axis) is based on the Rand index of the cluster identities compared with the true simulated cluster identities.

A soft threshold involves taking even powers of each element of the correlation matrix. Taking even powers gives negative correlations the same weight as positive correlations, but for simplicity we only simulate positive correlations. Higher powers force lower correlations closer to zero, reducing their role in the cluster analysis. For soft thresholds (Figures 6, 7), WGCNA has slightly higher Rand index accuracy than RIP-M and both have higher accuracy than Newman Modularity. The accuracy of RIP-M and WGCNA is flat across soft threshold powers, whereas Newman Modularity tends to increase in accuracy with increasing threshold.

### Influenza vaccine RNA-Seq results

To compare performance on real data, we applied RIP-M and WGCNA to an RNA-Seq co-expression network for an influenza vaccine experiment. Gene expression for subjects was measured before and 3 days after receiving influenza vaccine. We computed the log2 fold change between Days 3 and 0 for each subject's transcripts and then we removed transcripts showing low magnitude or variation across all subjects, which left 4956 transcripts. We calculated the Pearson correlation between all pairs of transcripts across all 105 subjects, and we applied the absolute value to the coefficients to treat activation and repression connections the same in the network.

**Figure 8:**
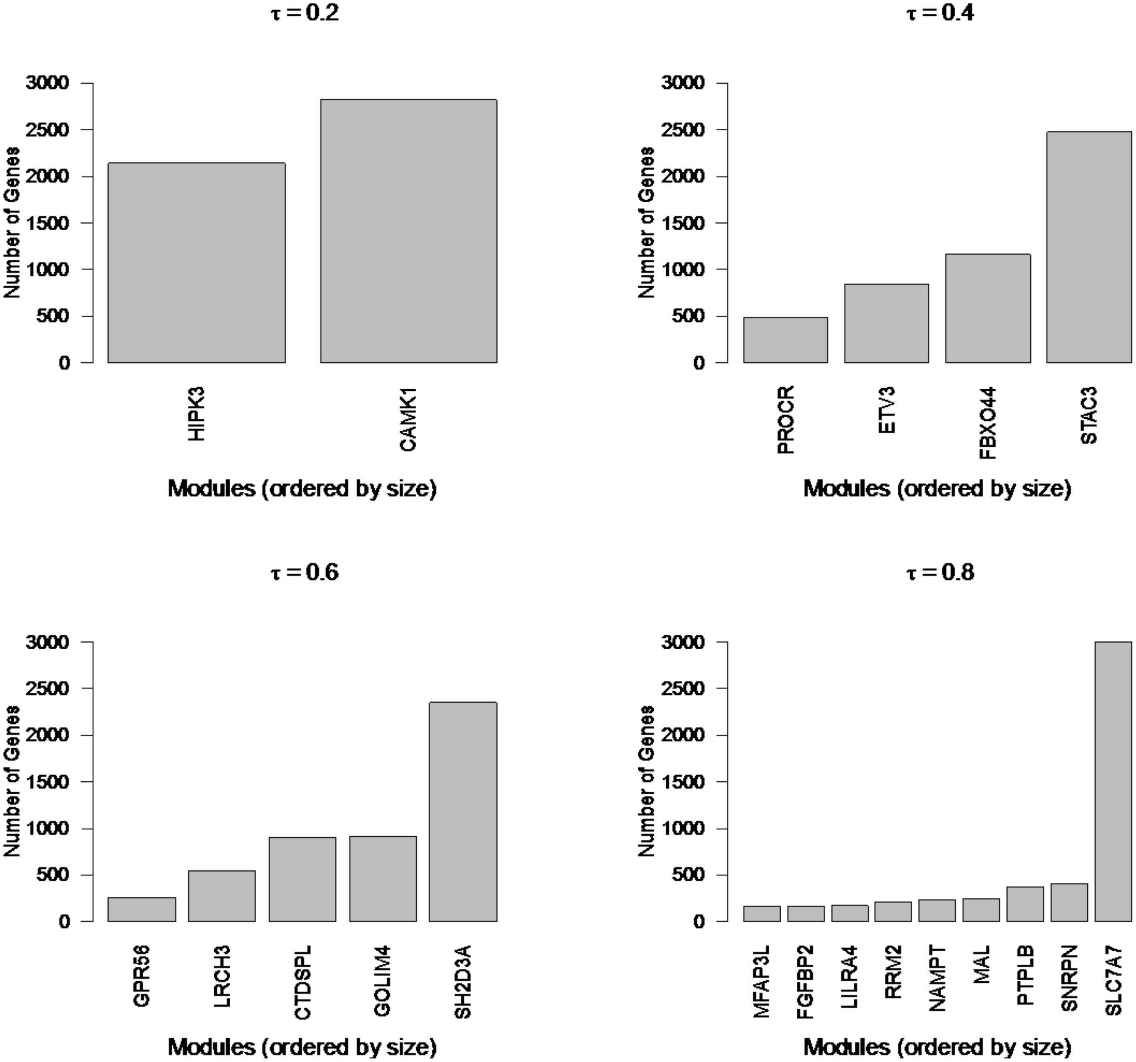
**Newman Modularity module sizes for flu vaccine RNA-Seq co-expression network for a range of hard thresholds (τ)**. The modules from each run (from 4965 transcripts) are labeled by their hub gene (gene with highest within module degree). Modularity finds large components for each value of τ.

**Figure 9:**
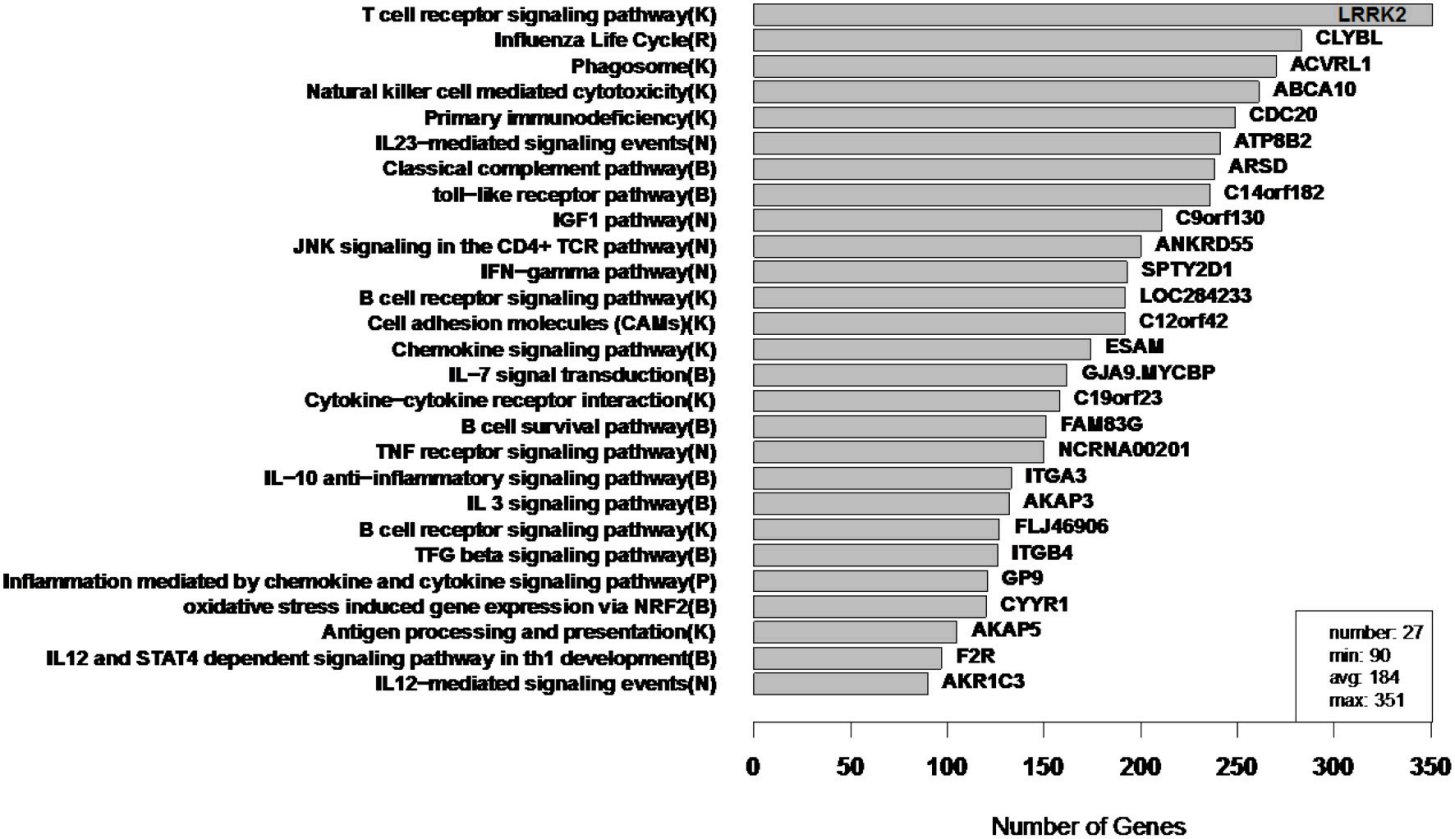
**RIP-M module sizes for flu vaccine RNA-Seq co-expression network**. Each of the 27 modules (from 4965 transcripts) is labeled by its most enriched immune system pathway (on the left) and by its hub gene (gene with highest within-module degree) on the right. Hard threshold τ = 0.2 of the absolute value of Pearson correlation was selected. The hard threshold choice was guided by the goal of having modules without extreme sizes, with a minimum size of 100 and maximum of 300. Parentheses in pathway names indicate source of annotation (Reactome, KEGG, NCI-PID, and BioCarta).

**Figure 10:**
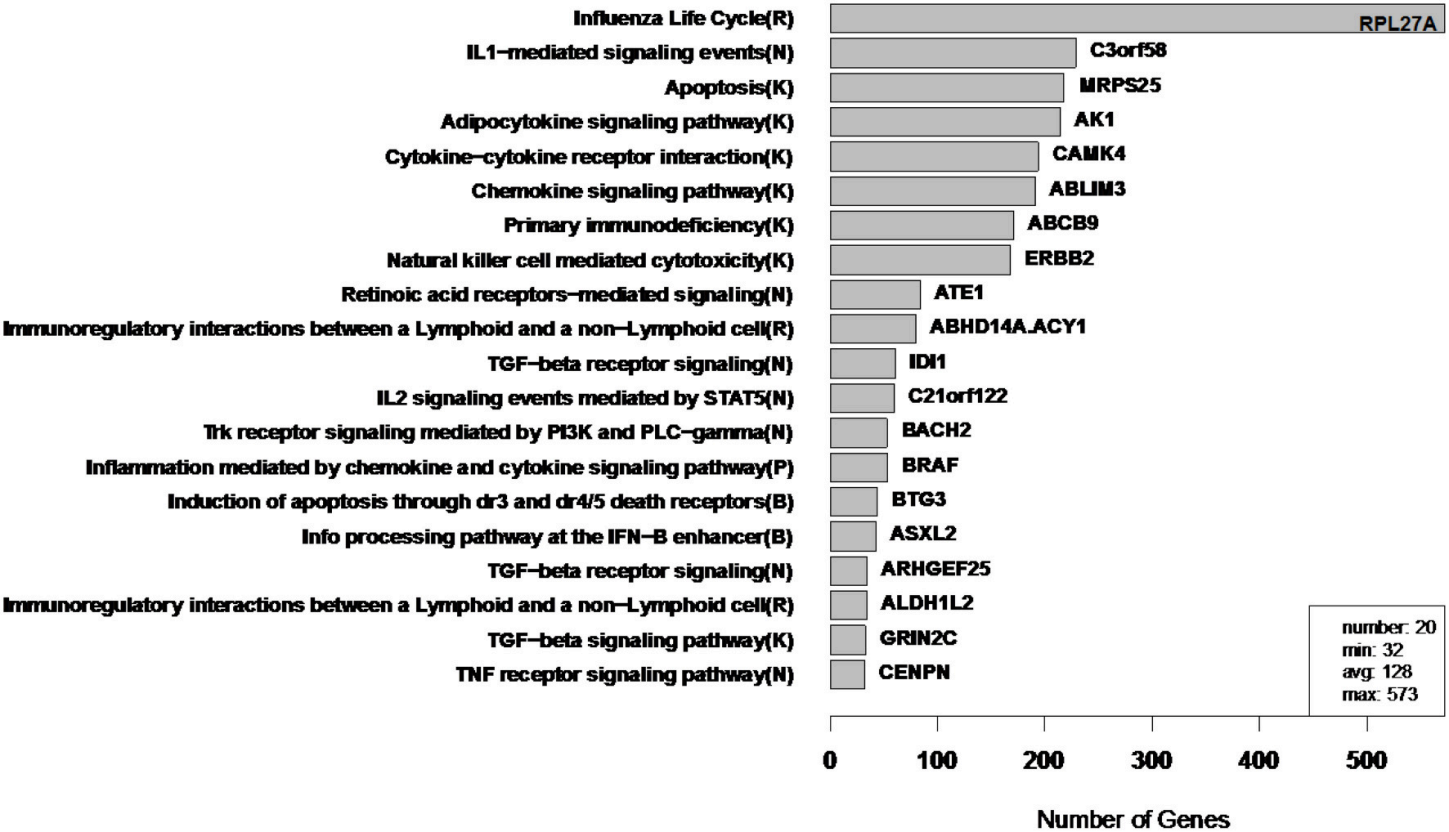
**WGCNA cluster sizes for flu vaccine RNA-Seq co-expression network**. Each of the 20 clusters (from 4965 transcripts) is labeled by its most enriched immune system pathway (of the left) and by its hub gene (gene with highest within module degree) on the right. These clusters contain 2543 of the original 4965 because 2422 fell into the “unassigned” cluster. Algorithm parameters and hard threshold were swept with the goal of finding a module size distribution without extremely small or large modules. Parentheses in pathway names indicate source of annotation (Reactome, KEGG, NCI-PID, and BioCarta).

To illustrate the types of extremes that can be observed in cluster sizes, we applied Newman Modularity to the RNA-Seq network for a range of hard threshold values (τ). For each value of τ, Modularity finds a giant component (Figure 8), which was the original motivation for the RIP-M modifications. We then swept the hard threshold parameter (τ) over a range of settings to identify clusters for RIP-M and WGCNA with limited small and large components. We specified a minimum cluster size of 100 and maximum of 300, with the goal of obtaining approximately 25 uniform clusters (average size of 200). This target was not quite achieved for RIP-M or WGCNA (Figures 9, 10), but these hard thresholds yield the closest approximations. The WGCNA cluster sizes are more extreme than RIP-M, but of course the true size distribution is unknown. We provide rationale for choosing these clustering parameters in the Discussion.

In order to assess the biological relevance of the RIP-M and WGCNA influenza RNA-Seq clusters, we tested geneset enrichment of known immune system and other functional pathways that we predict may be related to vaccine response. We used the Reactome Functional Interaction (FI) database to identify enriched biological pathways in the genesets defined by each cluster (Vastrik et al., 2007). We calculated the fraction of overlap of each module with immunological gene sets and used the hypergeometric *p*-value to quantify significance of enrichment. We mapped each RIP-M module (Figure 9) and WGCNA cluster (Figure 10) onto the most enriched immunological gene set and sort them by cluster size. For example, “Influenza Life Cycle(R)” is enriched in one of the larger partitions of both methods, and “Cytokine-Cytokine Receptor” is enriched by both methods in more intermediate-sized clusters. To look beyond only the most immunological pathway in each cluster, we used a q-value cutoff of 0.1 for all RIP-M modules and WGCNA clusters. With this threshold, we identified 27 immunological gene sets enriched by both algorithms. The RIP-M modules are enriched for an additional 17 immune gene sets that are distinct from WGCNA clusters, and WGCNA clusters are enriched for 10 immune gene sets that are distinct from RIP-M. This suggests that additional biological insights may be obtained through a combined clustering approach.

**Figure 11:**
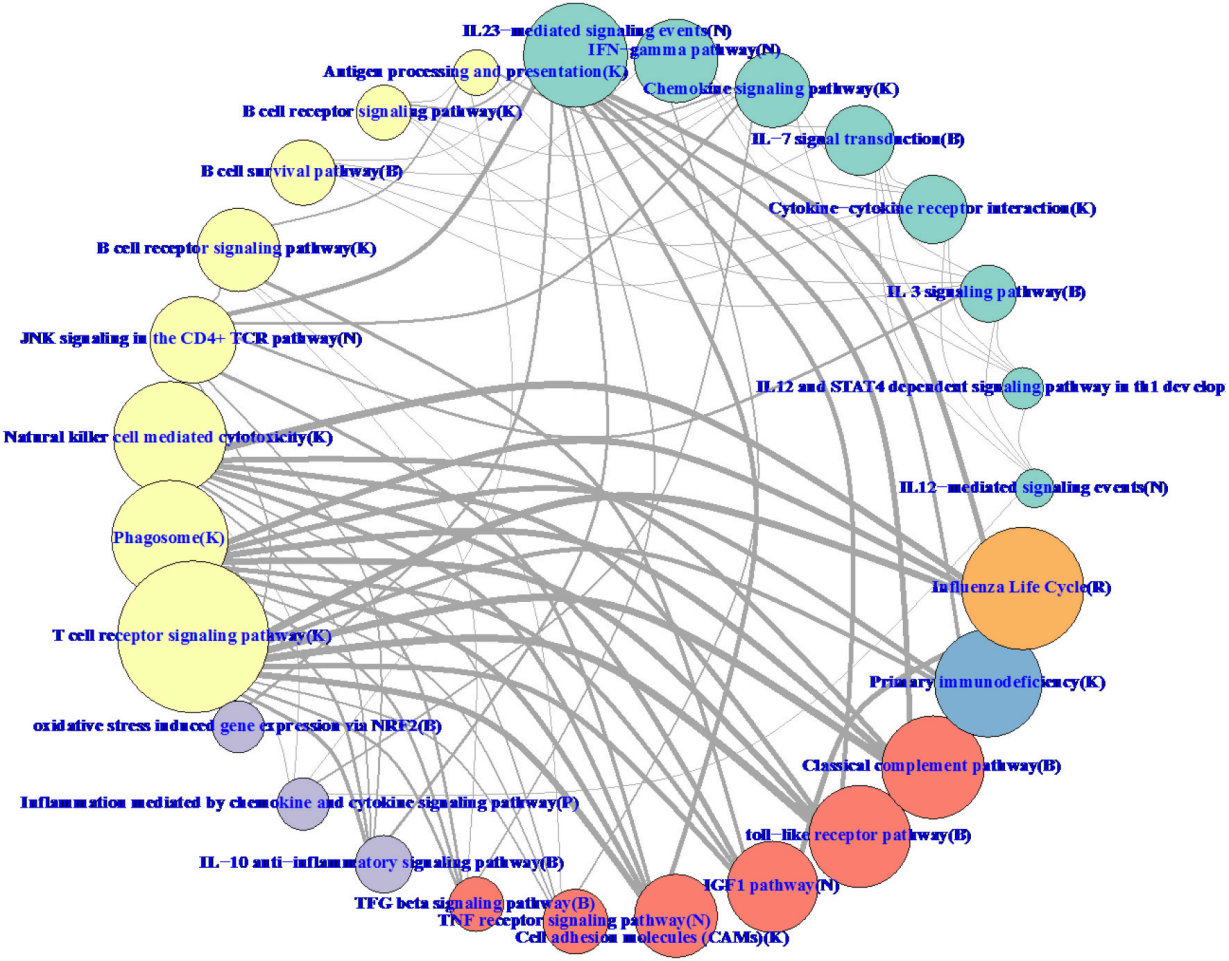
**RIP-M pathway-collapsed network for RNA-Seq influenza vaccine data with 4965 transcripts showing highest variation**. For each of the 27 RIP-M modules, genes and their within-module edges are collapsed into one node and labeled by its top immune system pathway. The radius of each node is proportional to the number of genes in the module. The weight of each edge is the total number of edges between genes of the two modules. The top five connections are shown for each module for clarity. The modules were obtained by hard threshold τ = 0.2 of the absolute value of Pearson correlation. Colors were chosen based on pathway-type: yellow T-cell, B-cell, NK-Cell; green cytokine; purple inflammatory; blue immunodeficiency; orange influenza life cycle; red TLR and complement.

Hierarchical methods like WGCNA have a natural tree-based visualization of the relationships between genes. With RIP-M, it is more natural to visualize the relationships as a network graph (Figure 11). To balance the volume of information displayed on the graph with its interpretability, we collapsed the individual gene connections within a module onto one node and label the nodes based on their most enriched immune system pathway. Edges between collapsed nodes are weighted based on the number of edges between genes in each module. The aggregation of genes within modules/pathways and aggregation of the connectivity may increase sensitivity to detect regulation between pathways. For example, IGF-1 has been shown to promote cytotoxic activity in human natural killer cells (Ni et al., 2013), and our influenza vaccine pathway network (Figure 11) shows a strong connection between “IFG1 Pathway” and “Natural killer cell mediated cytotoxicity.” Connections at the individual gene level within the two pathways may miss this relationship.

## Discussion

RIP-M is an extension of Newman Modularity for community detection that provides additional flexibility to identify substructure in modules that may be more relevant to the application domain. We compared and assessed this approach to find functional communities of genes in RNA-Seq gene co-expression networks; however, RIP-M is not limited to this application domain. We extended Newman Modularity to operate on indirect-path networks (IP-M) in order to increase information flow between nodes in the network and thereby merge modules with sizes that fall below a user specified size, below which biological pathway enrichment may be difficult to detect. Instead of using only the adjacency matrix, A, in the modularity B matrix, we use a power series of A (Equation 4), which represents higher order indirect connections between genes. Incorporating these indirect connections has the effect of merging smaller modules together into more reasonable sizes. The merging process, however, may leave large network components that contain unresolved substructure that have more specific biological function. Thus, we combined the IP-M merging capability with a splitting algorithm to decompose large modules and resolve finer functional pathways. We balanced the merging and splitting operations recursively in a modified version of modularity we call Recursive Indirect-paths Modularity. We compared this algorithm with standard modularity and hierarchical WGCNA on simulated correlation networks and real RNA-Seq co-expression from an influenza vaccine study.

It is common practice to use hard and soft thresholds to remove noise (reduce false-positive edges) from co-expression networks. We find that thresholds can also affect the number and size of clusters. A byproduct of this noise reduction is that some true direct connections may be lost (increase in false-negative edges). Using RIP-M, evidence for connections between nodes may be recovered from the adjacency matrix through evidence from indirect connections. Some pairs of genes that fall below the correlation threshold due to weak experimental or biological signal may show stronger indirect support for being linked based on evidence from the number of shared neighbors. We show that including higher-order connections in the generalized indirect-paths modularity has a merging effect on modules; some connections between nodes are strengthened and nodes from smaller clusters are rearranged into larger clusters.

The results on simulated and real co-expression data suggest that RIP-M has some advantages over Newman Modularity, on which RIP-M is based. For hard and soft thresholding of the simulated correlation matrices with homogeneous and heterogeneous cluster sizes, RIP-M and WGCNA have higher accuracy than Newman Modularity for finding the correct number of communities and correct partition identities of the nodes. In the simulated and real RNA-Seq co-expression data, Modularity has a tendency to create partitions with a small number of communities with some giant components, compared to WGCNA and RIP-M, which tend to resolve the co-expression networks into more intermediate sized clusters. WGCNA and RIP-M find comparable cluster numbers for simulated and real data. For the real data, WGCNA and RIP-M modules enrich for many of the same immunologically relevant pathways. Both methods enrich for some distinct immunological pathways, and there may be an advantage to taking the union of enriched pathways by both methods to carry forward for additional statistical analysis.

With the expectation that genes involving the immune system will be most stimulated by vaccination, we compared methods based on enrichment of communities of genes in immune functional pathways. RIP-M and WGCNA communities were enriched with the majority of the same immunological pathways, and each method found some unique pathways. Replication studies of these pathways will lead to a better understanding of the clustering mechanisms that lead to biologically useful communities and whether a hybrid approach may take advantage of unique mechanisms of each approach. We used enrichment of immunological pathways in the Reactome FI database; however, these pathways involve complex signaling and other interactions, and their genes are thus likely to have connections to multiple pathways, not simply connections within one pathway. This complexity adds to the noise of identifying partitions in this gene expression experiment, and in general one expects gene cluster membership to be fuzzy. Future work should involve testing for enrichment in other biological studies where the gold standard pathways may be more circumscribed or includes less heterogeneity, such as model organisms.

RIP-M and WGCNA allow the user to constrain cluster sizes. In this sense, RIP-M is similar to clustering algorithms that have the number of clusters as an input, like WGCNA, which may be suboptimal for data where cluster sizes fall outside the range. However, RIP-M and WGCNA are permitted to return cluster sizes outside of the user specified range (remaining true to actual relationships within the data). We simulated homogeneous and heterogeneous-sized clusters, where some cluster sizes fall outside the specified range, and RIP-M retains high accuracy. If no cluster size preference is provided (a wide range of cluster sizes is used by default), the RIP-M algorithm will still look for indirect evidence for connectivity and attempt to perform merging and splitting to find the best partition of the genes according to the relationships present within the supplied data. In the simulation studies, RIP-M is able to accurately find modules when the underlying data is composed of either heterogeneous or homogeneous cluster sizes.

Algorithm parameter selection is an important issue in clustering that is not unique to RIP-M, as WGCNA has a similar number of parameters that may be adjusted. In the real data, our choice of τ = 0.2 was influenced by simulation results (Figures 4, 5), but more by the fact that it resulted in clusters with the most uniform sizes. Increasing τ in the real data did not have a drastic effect on the number of clusters. We chose 25 as our target number of clusters because, of the roughly 5000 filtered genes, uniform clusters would contain approximately 200 genes each, which is a common number for geneset enrichment analysis. For example, the C7 immunologic signatures in MSigDB use geneset sizes of 200 (Godec et al., 2016). The C7 genesets correspond to top or bottom genes (FDR < 0.25 or maximum of 200 genes) for each comparison of conditions in immunologic gene expression experiments. In Chaussabel et al. the authors used *k* = 30 in k-means clustering of the coordinated gene co-expression across multiple immune phenotypes to create modules of genes suggestive of coordinated biological activity (Chaussabel et al., 2008). WGCNA recommends setting hard and soft thresholds based on scale free degree distribution, but, like our approach, this may lead to undesirable cluster assignments of genes. After deciding on a target of 200 genes, we then chose minimum and maximum cluster size parameters 100–300 in RIP-M because this range brackets the average module size target. Using different RIP-M parameters resulted in similar final cluster compositions. Our reported clusters with the choice of τ = 0.2 had the most uniformly distributed sizes.

Another application of gene partitioning into modules is to use the collective information as a feature in a machine learning classifier of a phenotype or outcome. It has been shown for genetic association studies of multi-genic disorders that associations tend to be found in similar functional pathways (Franke et al., 2006). This suggests that constructing an attribute from the genes in a module or functional pathway may improve the ability to predict phenotypic class or variance while also reducing the multiple-hypothesis burden. Future study is needed to assess the potential advantage of combining clustering approaches with attribute construction approaches, such as single subject GSEA, GSVA (Hanzelmann et al., 2013) or within-cluster hubs, for use as attributes in machine learning algorithms to improve their ability to predict disease susceptibility, immune response or other relevant outcomes. One may also combine RIP-M or other clustering methods with outcome information through differential co-expression analysis (Lareau et al., 2015).

We have extended the Newman Modularity method for community detection by including indirect-path information when computing modules and by creating the RIP-M merge-split algorithm that provides additional flexibility to identify network substructure. We tested RIP-M on synthetic and real RNA-Seq gene co-expression networks, identifying interpretable and biologically descriptive modules. For example, our results show a clear concentration of gene expression activity in several complementary areas of biological function important for both innate and adaptive immunity. The day 3 expression data delineates connections between several major pathways including JNK signaling in CD4 T cells and T cell receptor signaling, or B cell receptor signaling and B cell survival. It also illuminates the complex interplay between different immune processes such as IL-23 signaling (driving Th17 responses); IFNg, TNFa, and IL-12 (driving Th1 responses); anti-inflammatory signaling through TGF-b, IL-10, and the combination of IL-3 and IL-7 signaling known to drive hematopoietic production of lymphoid progenitor cells in both mice and humans (Grabstein et al., 1993; Puel et al., 1998; Crooks et al., 2000). RIP-M shows higher accuracy than Newman Modularity, and RIP-M's performance is comparable to WGCNA. Like WGCNA, RIP-M allows calibration of cluster sizes, but without removing genes. We believe RIP-M will be a valuable addition to the tools of computational biology for finding context-specific sets of genes for further pathway and integrative analysis.

## Availability

The raw data used in this study is available on ImmPort at https://immport.niaid.nih.gov/ under study number SDY67. Code for RIP-M and the normalized data used in this study are available at our website: http://insilico.utulsa.edu/index.php/ripm/.

## Author contributions

BR, BW and BM conceived of the method and performed analyses. BR and BM wrote the initial draft of the paper. MZ, DG, GP, RK, and AO contributed to the design, interpretation, and revisions for content. All authors approved the final version and agree to be accountable to the accuracy and integrity of the work.

## Disclaimer

Dr. GP is the chair of a Safety Evaluation Committee for novel investigational vaccine trials being conducted by Merck Research Laboratories. Dr. GP offers consultative advice on vaccine development to Merck & Co. Inc., CSL Biotherapies, Avianax, Dynavax, Novartis Vaccines and Therapeutics, Emergent Biosolutions, Adjuvance, Microdermis, Seqirus, NewLink, Protein Sciences, GSK Vaccines, and Sanofi Pasteur. Dr. GP holds two patents related to vaccinia and measles peptide research. These activities have been reviewed by the Mayo Clinic Conflict of Interest Review Board and are conducted in compliance with Mayo Clinic Conflict of Interest policies. This research has been reviewed by the Mayo Clinic Conflict of Interest Review Board and was conducted in compliance with Mayo Clinic Conflict of Interest policies.

### Conflict of interest statement

The authors declare that the research was conducted in the absence of any commercial or financial relationships that could be construed as a potential conflict of interest.
